# Supplementary material for: Improved Tumor Blood Flow Enhances the Abscopal Effect: Preclinical Assessment in Mice Treated with Combined Radiation and PD-1 Blockade Therapy
Source: bioRxiv. 2025 May 9:2025.05.04.652150. Preprint. [Version 1] doi: 10.1101/2025.05.04.652150 (PMC12190758; doi:10.1101/2025.05.04.652150)
Supplement: Supplement 1 [file media-1.docx]

A

B

Supplementary Figure 1: Correlations between (A) DCE AUC 10min and (B) EPR Hypoxic Fraction on the remote tumor on day 0 and remote tumor volume on day 9.
